# Observations Respecting a New Mode of Treatment in Glanders, Farcy, and Obstinate Grease

**Published:** 1807-12

**Authors:** John Roberton

**Affiliations:** Surgeon, Edinburgh; Author of the Practical Treatise on Cantharides, in Gleet, Leucorrhœa, and Sores; and Extraordinary Member of the Royal Medical Society


*Observations respecting a New Mode of Treatment in Glanders, Farcy, and Obstinate Grease.* By JOHN ROBERTON, Surgeon, Edinburgh; Author of the Practical Treatise on Cantharides, in Gleet, Leucorrhœa, and Sores; and Extraordinary Member of the Royal Medical Society.

IT is to be regretted that the Diseases of the Horse should have hitherto met with so little attention, and received so little benefit from the labours of scientific men. Their researches have often been directed to subjects of much less importance, even if the objects of these researches had answered their most sanguine expectations. I am, however, sorry to say, that without their being, for the most part, even conscious of what they wished to establish, brutal and unmeaning experiment upon living animals has too often occupied their time, while the diseases of this noble creature have been utterly forgotten; or, what is worse, left solely to the mercy of the murderous quackery of ignorant Farriers and Grooms; men who, from their situation in life and the nature of their profession, could not be expected to possess any knowledge of the operations going forward in the animal machine, either in a state of health or disease. Such men, however, have been permitted to superintend the treatment of every variety of disease, to which the Horse is liable. Influenced by the grossest prejudice, they, by the indiscriminate use of a few stale rules and ridiculously absurd prescriptions, have certainly been very successful in destroying the animal; but, from the information I have been able to acquire on that subject, their mode of treatment seems seldom to have served any other purpose.

As, for the most part, the diseases of the Horse are, particularly at their commencement, of an inflammatory nature, the only service these men ever did (and that, I believe, to have been more by accident than by observation, or the dictates of reason) was, on the approach of disease, to use the indiscriminate abstraction of great quantities of blood, or to administer brisk purgative medicines.

Now the consequences of inflammatory affections are always totally different from the primary symptoms; and, consequently, the treatment of each ought to be varied. These men, however, have treated all of them, in all their stages, nearly on the same principle. Much mischief has, therefore, been done to those useful animals; and, consequently, much loss has accrued to every country in which they exist.

Of late years, however, government has wisely appointed veterinary surgeons in the army (while private gentlemen have neglected the plan) which has, no doubt, contributed very much to the method of cure in these diseases, being conducted on more rational principles. This, of course, will in time be attended with greater success; these gentlemen being, in general, possessed of liberal education, and consequently uninfluenced by those prejudices which were inseparable from their predecessors—the farriers and grooms; and I have no doubt that, from their exertions, this branch of medical science will derive much benefit.

The books already published on this subject, both ancient and modern, seem to me very ill calculated to add to our information. What, therefore, we are to depend on, must be attention to the individual diseases of the animal; from which, I think, an entirely new, more rational, and useful system of veterinary surgery, may soon be obtained; as nothing of the kind, that can be relied on, at present exists.

The diseases of all animals, and more especially those of the same classes, I believe, nearly resemble each other in many particulars; and, if we reason from analogy, perhaps, without any material alteration, so far as regards general principle, that treatment which is applicable to diseases of the human species, may, in a great measure, be applied to those of the horse.

It was this mode of reasoning which led me to believe, some years ago, when I had been endeavouring to satisfy myself respecting the powers of cantharides, used internally on the living body, both in a state of health and disease, that many of those diseases of the horse, which were deemed incurable, might be alleviated, or entirely removed, by the judicious employment of this substance.

The

The same process of reasoning, which led me to employ cantharides in gleet, leucorrhœa, and ulcers, in greater doses, and for a much larger period than had hitherto been done*, without producing any of those dangerous consequences which prevented others from employing this medicine, and to which alone I attribute their want of success in the removal of these affections, convinced me that the properly regulated administration of them, in some diseases of the horse, would be followed by consequences equally beneficial, particularly in the diseases of Glanders, Farcy, and Obstinate Grease. Though attempts have been made to cure these diseases, none of the remedies at present in use, or the rules laid down by veterinary writers, can be depended on; particularly in glanders, where the disease is no sooner discovered than, from its infectious nature, the animal is instantly destroyed.

As leucorrhœa and gleet depend on a diseased action of the mucous membrane of the vagina and urethra, or of the glands situated immediately under them, in consequence of previous inflammatory action, so the disease of glanders in the horse, is a similar affection of the membrane of the nose; and as, in the former diseases, a complete cure may uniformly be effected, by the properly administered use of cantharides, so a cure of the latter, as well as of farcy and obstinate grease, may be effected by producing that degree of inflammatory action in the system, which is necessary to restore debilitated organs to their healthy function, and during which wounded surfaces are healed. So far, at least, as my limited opportunities have permitted me to judge, my encouragement has been such as to warrant the completest success.

I need not say any thing respecting the dose of cantharides for the horse, as in our own species; I have found it necessary to vary it, according to the severity of the disease and the constitution of the individual. I may, however, mention that, as a sort of general rule, about half a scruple of the powder made into a ball, may be given twice a day; gradually increasing the dose, or the same quantity thrice a day, to very debilitated animals; care being taken, at the same time, to watch the pulse, and, likewise, its effects on the urinary organs. If the last of these becomes distressing, the administration of a brisk purgative, or even general blood-letting; and, with either of these,

* See Treatise on Cantharides.

cloths cloths dipt in warm water, and applied to the belly, are sufficient to remove its effects.

To the horse I would prefer giving the powder of cantharides, rather than the tincture; as, in the form of a ball, we may more easily judge of the precise quantity actually swallowed, than we can by mixing it with soft meat.

In a subject of such national importance, it would yield me the greatest happiness, if the hints I have here thrown out, or any other exertion that lies in my power, should effect the completion of this undertaking.

I have long wished to institute a regular course of experiments on this subject; but, from professional engagements, and there being no regular veterinary college in this part of the country, I have not yet been able to put my plans in execution to the extent that I could wish; but I have communicated my views on that subject to several gentlemen, who may be in the habit of treating diseases of the horse, or may have acquaintances who have such opportunities; and all of them have obligingly and readily offered their assistance, in order to accomplish the object I have long had in view.

From the extensive circulation of your valuable Journal, I hope these remarks will be more widely known, than I can be expected to make them by any other means; and, if any gentleman will do me the favour to treat cases of the above description on my plan, and communicate the result to me, should they be successful, they will do their country an essential service.

*No. 4, St. James's Street,*
*October,* 1807.

